# B-Mode and Contrast Enhanced Ultrasonography Features of Gastric Inflammatory and Neoplastic Diseases in Dogs

**DOI:** 10.3390/ani11030670

**Published:** 2021-03-03

**Authors:** Francesco Simeoni, Francesca Del Signore, Giovanni Aste, Paolo Bargellini, Giuseppe Rubini, Rossella Terragni, Roberto Tamburro, Ilaria Falerno, Francesco de Pasquale, Marco Russo, Massimo Vignoli

**Affiliations:** 1 Faculty of Veterinary Medicine, University of Teramo, SP 18, 64100 Teramo, Italy; fdelsignore@unite.it (F.D.S.); rtamburro@unite.it (R.T.); ifalerno@unite.it (I.F.); fdepasquale@unite.it (F.d.P.); mvignoli@unite.it (M.V.); 2 Tyrus Veterinary Clinic, via Aldo Bartocci 1G, 05100 Terni, Italy; paolobargellini3@gmail.com; 3 UltraVet Diagnostic, via Enrico Fermi 59, San Giovanni Persiceto, 40017 Bologna, Italy; giusepperubini@ymail.com; 4 Pet Care Veterinary Clinic, via Marzabotto 1/2 M-N, 40133 Bologna, Italy; terragni.rossella@gmail.com; 5 Department of Veterinary Medicine and Animal Production, University of Naple, via Federico Delpino 1, 80137 Napoli, Italy; marco.russo@unina.it

**Keywords:** gastric neoplasia, gastric inflammation, b-mode ultrasound, contrast enhanced ultrasound, dogs

## Abstract

**Simple Summary:**

Canine gastric disorders are common in veterinary clinical practice and among these neoplasms require rapid identification and characterization. Standard ultrasonography is the imaging modality of choice for gastric wall assessment. The aim of this prospective study is to describe the specific B-mode and contrast enhanced ultrasound features of normal, inflammatory, and neoplastic gastric diseases in dogs. Standard and contrast enhanced ultrasound of the stomach were performed in anesthetized dogs with or without gastric disorders. Gastric wall qualitative and quantitative parameters were evaluated. A total of 41 dogs were included: 6 healthy as the control group; 9 gastritis; 8 adenocarcinoma; 8 alimentary lymphoma; 4 leiomyosarcoma; 2 gastrointestinal stromal tumor; 2 leiomyoma; 1 undifferentiated sarcoma; 1 metastatic gastric hemangiosarcoma. Gastric tumors appear as a marked thickening with absent layers definition and possible regional lymphadenopathy and steatitis while gastritis generally shows no/mild thickening and no other alterations on standard ultrasound. During contrast—enhanced ultrasonography, neoplasms show a higher and faster wash in if compared to that of gastritis. B-mode and contrast enhanced ultrasound assessment may be useful in the evaluation of canine gastric disorders for the distinction between gastritis and gastric neoplasms, even if there are no specific aspects able to discriminate between different tumors types.

**Abstract:**

Canine gastric disorders are common in veterinary clinical practice and among these neoplasms require rapid identification and characterization. Standard ultrasound (US) is the imaging modality of choice for gastric wall assessment. The aim of this prospective study is to describe the specific B-mode and contrast enhanced US (CEUS) features of normal, inflammatory, and neoplastic gastric wall in dogs. B-mode US and CEUS of the stomach were performed in anesthetized dogs with or without gastric disorders. Gastric wall qualitative and quantitative parameters were evaluated on B-mode US and CEUS examination. A total of 41 dogs were included: 6 healthy (HEA) as the control group; 9 gastritis (INF); 8 adenocarcinoma (AC); 8 alimentary lymphoma (AL); 4 leiomyosarcoma (LEIS); 2 gastrointestinal stromal tumor (GIST); 2 leiomyoma; 1 undifferentiated sarcoma; 1 metastatic gastric hemangiosarcoma. Gastric tumors appear as a marked wall thickness with absent layers definition and possible regional lymphadenopathy (AC and AL) and steatitis (AC) while gastritis generally shows no/mild thickening and no other alterations on B-mode US. On CEUS, neoplasm shows a higher and faster wash in if compared to that of gastritis. B-mode and CEUS assessment may be useful in the evaluation of canine gastric disorders in the distinction between gastritis and gastric neoplasms, even if there are no specific features able to discriminate between the different tumor histotypes.

## 1. Introduction

Gastric neoplasms are uncommon in dogs and represent less than 1% of all neoplastic lesions in this species [1,2,3]. Clinical presentation in dogs affected by these tumors may include non-specific progressive gastrointestinal signs like chronic vomiting, hematemesis, melena, anorexia, and weight loss [4]. Possible hematological alterations are panhypoproteinemia and anemia related to chronic gastro-intestinal blood loss and malabsorption, increased liver enzymes due to neoplastic infiltration of the liver, and paraneoplastic hypoglycemia reported in dogs affected by gastric leiomyoma and leiomyosarcoma [4,5,6]. Adenocarcinoma (AC) is considered the most common canine gastric neoplasia with a variable prevalence (50–90%) reported in several studies [1,7,8,9,10,11]. A greater incidence of this neoplasm was described in certain breeds, such as the Belgian shepherd dogs, Collies, and the Staffordshire bull terriers [10,12,13]. Alimentary lymphoma (AL) is rare in dogs and affects more commonly the small intestine than the stomach, although it is the most common gastric neoplasia in cats [14,15]. Gastric mesenchymal neoplasm reported in veterinary literature include leiomyoma, the most common benign tumor, and leiomyosarcoma (LEIS) [1,2,16]. Furthermore, thanks to immunohistochemistry, some types of mesenchymal tumors have been reassigned and classified as gastrointestinal stromal tumors (GISTs) originating from the interstitial cells of Cajal [17]. Other reported canine gastric malignancies are mast cell tumors, sarcomas, extramedullary plasmacytoma, and rarely gastric carcinoid [2,18,19]. Other non-neoplastic gastric lesions may have similar appearances to that of the wall’s neoplasia [20].

Ultrasonography (US) is the best imaging technique for the assessment of gastric wall in terms of thickness and layering and gastric masses are usually identified with standard B-mode US, with greater sensitivity than radiography [21]. Gastric neoplasia usually appears as a mild to severe wall thickening with complete loss of normal layering: the thickening can be focal or diffuse, symmetrical or asymmetric, associated or not to loss of motility [22]. Other reported findings associated with gastric tumors are: gastric ulceration with or without perforation, mainly associated with gastric AC and LEIS [1,18,22]; regional lymphadenopathy, with enlarged and heterogeneous lymph nodes, especially in patients affected by AL and AC, where they may have a “target” appearance with poorly defined echoic rim and a highly defined echoic center [22]. However, the different types of gastric tumors may have a similar appearance on US and final diagnosis can only be made by cytological or histological examinations.

Contrast-enhanced ultrasonography (CEUS) is a safe imaging modality based on administration of intravenous contrast medium (CM) that may improve the visualization of blood vessels and microvascular architecture on US [23]. This US technique allows to perform qualitative and quantitative vascular assessment of various organs in both physiological and pathological conditions in dogs and cats [3,24,25,26,27,28,29,30,31,32,33]. Nowadays, CEUS has proven its usefulness in the characterization of inflammatory and neoplastic diseases in veterinary literature [26,27,28,29,33,34,35,36,37,38,39,40,41]. Regarding the stomach, a recent study described qualitative and quantitative B-mode and CEUS features of gastric wall in healthy cats, cats affected by gastritis, and cats with low and high grade gastric AL, reporting that gastritis and low grade AL had large overlap of qualitative and quantitative parameters both on B-mode and CEUS, while high grade AL usually appears as a severe hypoechoich wall thickening with absent layer definition, high-contrast uptake, a specific enhancement pattern with “comb teeth-like” vessels (parallel curvilinear structures representing arterial branching within the gastric wall) and associated regional lymphadenopathy, and local steatitis [41]. Furthermore, quantitative parameters such as thickness and peak enhancement (PE) were found to be useful in the characterization of feline gastric infiltrates with significant diagnostic accuracy (AUC > 0.70) [41]. In dogs, only one study has been conducted to investigate the reliability of low-mechanical index imaging compared to Doppler flowmetry for the assessment of gastric mucosal blood flow in healthy dogs, demonstrating the feasibility of CEUS quantitative evaluation in this organ [42].

Additionally, in human medicine, CEUS technique was evaluated to assess gastric perfusion kinetics during gastritis and/or gastric cancers [43,44]. One study reported that gastric cancer showed a diffuse enhancement with delayed and lower PE compared to gastritis, without “comb teeth-like”, while this pattern was visible in most cases of gastritis [43]. In another recent study, qualitative and quantitative CEUS parameters were evaluated in patients affected by gastric adenocarcinomas, lymphomas, and GISTs, proving a strong correlation between gastric blood flow measured by the CEUS and measured by laser Doppler flowmeter [44].

The aim of this study is to assess the specific B-mode US and CEUS features of healthy, inflammatory, and neoplastic gastric wall in dogs.

## 2. Materials and Methods

### 2.1. Animals

B-mode US and CEUS of the stomach were performed in dogs with or without gastric disorders. This multicentric prospective study was approved by the Ethical Committee (Prot. 2016/0090753) and dog’s owners sign a written consent before submitting the patients to the examinations. Dogs were included according to: (1) healthy dogs as control group based on physical examination, serum chemistry profile, complete blood cell count, and abdominal US which had to be anesthetized for planned castration or spaying; (2) dogs with gastric disorders enrolled based on clinical gastric related signs (chronic vomiting), serum chemistry profile, complete blood cell count, US abdominal examination, cytological or histological diagnosis of the gastric wall. All the patients included were fasted for at least 12 h before each study. Furthermore, all dogs included performed the examinations under general anesthesia to minimize motion artifacts (caused by polypnea, stress, and containment) to which the gastric wall is subjected especially in awake patients. Absence of clinical and US gastric disorders were confirmed in dogs enrolled as control group on a follow-up of 12 months after the first B-mode and CEUS examination. The inclusion of a healthy control group was considered crucial in the design of the study since no article describing the CEUS features of the canine gastric wall is present to date in veterinary literature with the same modalities described by us in terms of choice of contrast medium, analysis software, anesthesiology protocol, qualitative and quantitative analysis.

### 2.2. Anesthesiology Protocol

A 20 G catheter was inserted into the cephalic vein. The patients underwent the following anesthetic protocol: sedation with butorphanol (Dolorex^®^, MSD A.H., Madison, NJ, USA; 10 mg/mL), 0.3 mg/kg intramuscular, induction with propofol (Propovet^®^, Abbott Laboratories, North Chicago, IL, USA; 10 mg/mL) intravenous, followed by tracheal intubation and maintenance with an Isoflurane/O_2_ mixture. Vital parameters were monitored during the assessment.

### 2.3. B-Mode US Examination

Included patients were prepared for US. Once the abdomen was clipped and acoustic gel was applied, a conventional US of the abdomen was performed in right lateral recumbency. All the US were performed using a linear 7–12 MHz transducer on a dedicated machine (MyLab 70 XV, MyLab Twice and MyLab Eight, Esaote SpA, Genoa, Italy, Logiq E9 and Logiq S8, GE Healthcare Italy, Milan, Italy) with specific software for contrast ultrasonography. In dogs with no evidence of focal gastric lesions on B-mode examination, the CEUS study was performed at the level of the gastric body, while in patients with gastric lesions, it was performed in that specific area. Quantitative and qualitative data were collected for both the US techniques. Gastric wall thickness was measured, between the serosa and lumen interface, avoiding rugal folds, in dog with uniform gastric wall thickness, and at the maximum thickness area in dogs with transmural lesions or masses. Gastric wall and perigastric region were evaluated according to the following B-mode qualitative features: (1) layers definition, classified as normal, reduced, absent or pseudolayering (a moderately echogenic zone surrounded by an outer and inner poorly echogenic lines as described by Penninck et al. in 1998) [22]; (2) thickening, classified as absent, focal, or diffuse; (3) lesions echogenicity, classified as hyperechoic, hypoechoic, isoechoic; (4) regional lymph nodes, classified as normal or enlarged; (5) steatitis, classified as present or absent; (6) effusion, classified as present or absent. This B-mode examination protocol is very similar to that recently described and published for feline gastric disorders [41].

### 2.4. CEUS Examination

A second-generation intravascular CM (Sonovue^®^, Bracco Imaging Italia srl, Milan, Italy) was injected into the cephalic vein catheter (dose: 0.03 mL/kg) followed by a bolus of 1 mL saline solution using a three-ways valve to avoid any delay between the injection of CM and saline. Three contrast injections were performed in each patient. The first injection was used to perfuse the blood vessels and to fine-tune the machine setting. Second and third injections were performed for gastric wall perfusion studies: the second one was used for CEUS analysis; the third one was used instead of the second only in cases where the quality of the second scan was considered insufficient due to excessive gas and/or motion artifacts. Between the successive injections, to avoid artifacts, the remaining microbubbles were destroyed by modifying the acoustic power at the highest level and scanning the kidney, aorta, liver, and spleen for a few minutes. The basic technical parameters used were single focus, medium persistence, mechanical index 0.09 (40 kPa) and timer started at the end of the injection. For setting the gain, the starting point was a black/anechoic image, which represents the almost total suppression of the fundamental signal. The gain was optimized during the first injection to achieve a uniform image brightness. These settings have been repeated in the second and third injection. All studies were recorded for 90 s and digitized as a movie (clip) at a rate of 10 frames per second. The CEUS protocol reported above is the same as performed and published in our study on feline gastric disorders [41]. The clips were analyzed using a dedicated software (VueBox^TM^,V 7.0 version, Bracco Imaging Italia srl, Milan, Italy) for quantitative analysis. A region of interest (ROI) was manually drawn by the same operator based on the appearance of the gastric wall. In dogs with no evidence of gastric lesions on standard US, the ROI was drawn into the gastric wall between the serous and the mucosal interface, avoiding the gastric folds and large vessels. If dogs with US evidence of gastric wall thickening, the ROI was drawn within the lesion or mass. To optimize the CEUS quantitative analysis, the motion compensation software of VeuBox^TM^ was applied to minimize the effect of respiratory movements on the displacement of the ROI from the gastric wall. For each ROI, the software determined the average pixel intensity and created a time-intensity curve, which was subsequently analyzed to calculate different blood flow quantitative parameters: blood volume, expressed by PE and wash-in rate (WiR) in decibels (dB); blood velocity, expressed by arrival time (AT), rising time (RT), time to peak (TTP) and time to fall (TO), all in seconds (s). For each group of values and each study, means and standard deviations (SD) were calculated and reported. The enhancement patterns of gastric walls considered are the same as those reported by Xue et al. in the human medicine literature and by Simeoni et al. for gastric disorders in cats [41,43]: (1) enhancement degree, considering visible normal liver parenchyma as the reference. The relative enhancement of the lesion at contrast arrival was classified as hyper-, iso/hypo-, or non-enhancing; (2) homogeneity of contrast uptake, classified as homogeneous or heterogeneous enhancement; (3) comb teeth-like vessels, consisting of parallel curvilinear structures representing arterial branching within the gastric wall on CEUS, classified as present or absent. Enhancement of these vessels can only be seen in the early arterial stage and usually last for about 1 s before being immersed in the diffused enhanced gastric wall. Because of this relatively short time window, CEUS clips have to be replayed frame by frame to determine their presence [41].

### 2.5. Characterization of Pathological Gastric Wall

To determine the nature of the gastric disease, for each dog, the gastric wall was sampled, guided by US or by endoscopy, and analyzed by cytological or histological examination for characterization and classification.

### 2.6. Statistical Analysis

The statistical evaluation was performed using the *jamovi* software (V.1.2) (Sidney, Australia). Differences in the distribution of qualitative B-mode parameters among the different groups were tested with the Fisher’s exact method. The differences in the distribution of the quantitative parameters in relation to the different groups were analyzed using a one-way analysis of variance (ANOVA) for normally distributed data or with the Kruskal–Wallis test for non-normally distributed data. A value of P less than 0.05 was considered statistically significant for each test.

## 3. Results

### 3.1. Study Population

A total of 44 dogs underwent B-mode US and CEUS examination between 2017 and 2020 and related data were collected during the same period. No adverse effects were noted in any patients. Because of the poor clip’s quality obtained from the CEUS examination, 3 dogs were excluded. A total of 41 dogs were included in the study and were divided into the following groups: 6 healthy (HEA) in the control group (mean age 4.1 ± 2.3 years, 4 male and 2 female), 9 gastritis (INF) (mean age 5.33 ± 3.5 years, 7 male and 2 female), 8 AC (mean age 11.88 ± 2.1 years, 2 male and 6 female), 8 AL (mean age 10.13 ± 1.8 years, 4 male and 4 female), 4 LEIS (mean age 13 ± 2.4 years, 2 male and 2 female), 2 leiomyoma (mean age 12.5 ± 0.7 years, 2 male), 2 GIST (mean age 9.75 ± 3.2 years, 2 female), 1 poorly differentiated sarcoma (9 years, male), and 1 metastatic gastric hemangiosarcoma (10 years, male). All the HEA dogs included in the control group were followed up on and checked 12 months after the first examination, confirming the absence of clinical and ultrasonographic signs of gastric disease. Statistical analysis was performed among the following groups: HEA, INF, AC, AL, and LEIS. Pathological groups consisting of 2 or less dogs were not included in the statistics but were reported only descriptively.

### 3.2. Analysis of B-Mode Examination

**Figure 1:**
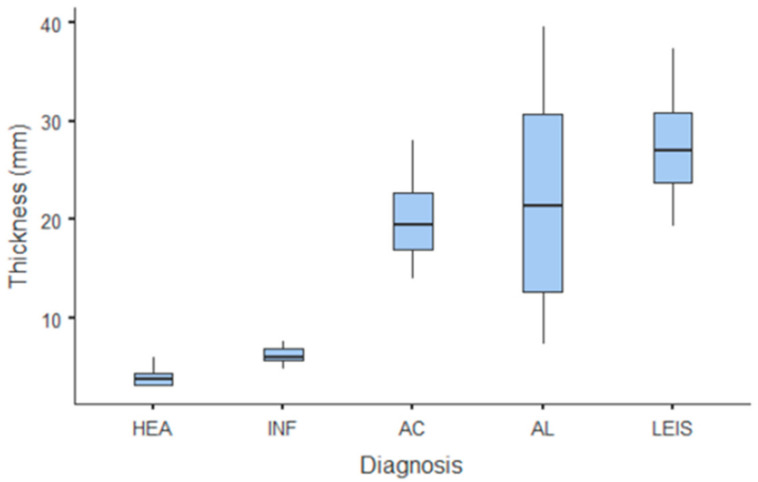
Box and whisker plot of the gastric wall thickness for healthy (HEA), inflammatory (INF), adenocarcinoma (AC), lymphoma (AL), and leiomyosarcoma (LEIS).

The only quantitative parameter considered in B-mode US was gastric wall thickness. Values and summary statistics of measured thickness with pathological classification are reported in Figure 1 and Table 1. Wall thickness means of each of HEA and INF groups were significantly lower than those of neoplastic groups (*p* = 0.002 vs. AC and *p* < 0.001 vs. AL and LEIS both); however, no statistically significant differences were observed between the thickness of HEA vs. INF, nor between any other of the considered neoplastic groups. For smaller pathological groups, mean thickness values are: 14.1 mm (±1.84 SD) for GIST; 11.3 mm (±2.62 SD) for leiomyomas; 40.5 mm for the undifferentiated sarcoma; and 21.1 mm for the metastatic hemangiosarcoma.

**Figure 2:**
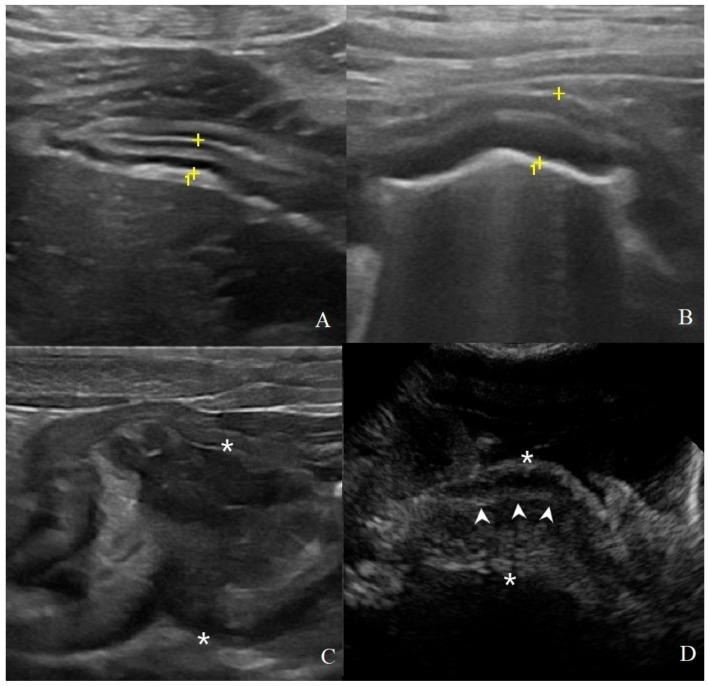
Classification of gastric wall layer definition on B-mode US: (**A**) normal: all the five layers are easily identified and thickness is normal, (4.1 mm between the cursors (+) in a HEA dog); (**B**) reduced: the identification of the layers is more difficult; in addition to a mild diffuse wall thickening (6.5 mm between the cursors in a INF dog); (**C**) absent: it is not possible to recognize the normal wall stratification and layer definition is lost. Here, a focal transmural mass of 37.5 mm between the asterisks in a gastrointestinal stromal tumor (GIST) with mixed echogenicity; (**D**) pseudolayering: wall, between the asterisk (*), with moderately echogenic line (arrowheads) surrounded by an outer and inner poorly echogenic areas in a dog with gastric adenocarcinomas.

**Figure 3:**
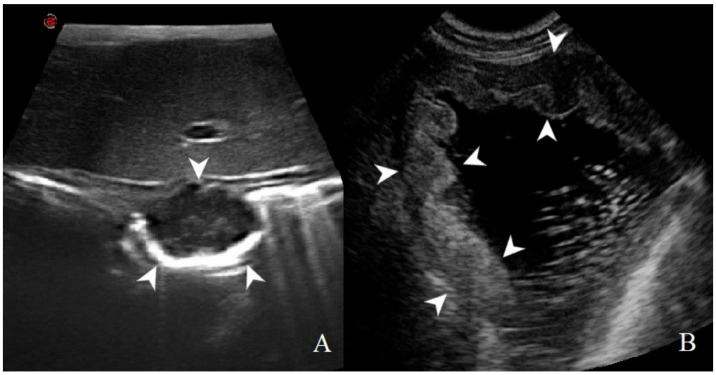
Thickening localization classified as: (**A**) focal: a transmural, prevalent hypoechoic mass (arrowheads) with loss of layering was identified in a dog with gastric GIST; (**B**) diffuse: wall hyperechoic thickening (arrowheads) with complete loss of layers definition is present in most of the stomach in this dog affected by adenocarcinoma.

Concerning the B-mode qualitative analysis (Table 2), significant differences were found in the wall’s layer definition classification (Figure 2) between HEA and the neoplastic groups (*p* < 0.05); HEA dogs had no alteration in layers definition (0/6). Additionally, INF showed the same differences of HEA if compared with the tumors groups (*p* < 0.05), although the gastric wall during gastritis may showed normal layers (5/9) or diffuse reduced layers definition (4/9). Otherwise, gastric neoplasia showed complete loss of wall’s layers (AC 6/8, AL 8/8, LEIS 4/4); part of AC (2/8) presented a “pseudolayering” aspect at the site of the gastric mass. Wall thickening localization (Figure 3), when present, were diffused for INF (9/9), while most gastric tumors present a focal thickening or mass (AC 7/8, AL 6/8 and LEIS 4/4). Lesions echogenicity differ significantly between the neoplastic groups (*p* < 0.001): the majority of AC (4/8) showed a hyperechoic lesion, while most AL showed a mixed echogenicity mass with heterogenous aspect (5/8), while half of LEIS have an hyperechoic mass and the other half a mixed echogenicity lesion. Lymphadenopathy, characterized by rounded lymph nodes, was found only in half of AC (4/8) and majority of AL (3/5), while no alteration in regional lymph nodes were found in HEA, INF and LEIS. As for the perigastric region, steatitis was found in the majority of AC (6/8) and some of AL (3/8) and LEIS (1/4) and was absent in HEA and INF groups. Furthermore, regional effusion was found only in AC and AL (1/8 for both). The 2 GIST were both focal mass; 1 was a focal hypoechoic lesion with loss of layers definition and 1 was a transmural lesion with mixed echogenicity, loss of layering definition with no other alteration of the surrounding structures. The 2 leiomyomas were focal hypoechoic masses with loss of layering without signs of lymphadenopathy, steatitis, or effusion. For the other neoplastic gastric lesions, B-mode qualitative features were: focal hypoechoic mass with absent layers definition and regional steatitis for the undifferentiated sarcoma; a focal mixed echogenicity thickening arising from the submucosal layer for the metastatic hemangiosarcoma.

### 3.3. Analysis of CEUS Examination

**Figure 4:**
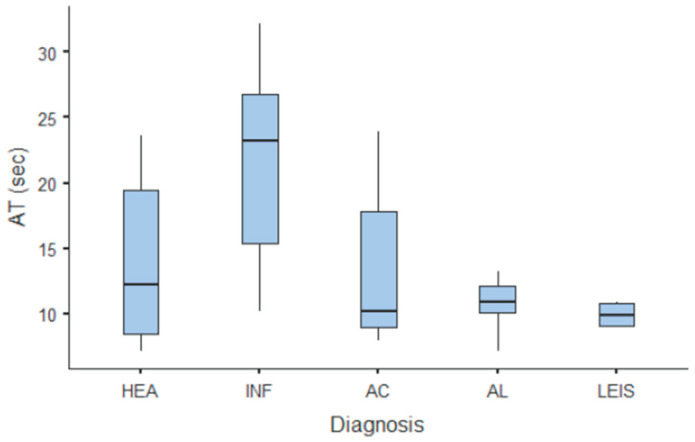
Box and whisker plot of the arrival time (AT) for healthy (HEA), inflammatory (INF), adenocarcinoma (AC), lymphoma (AL), and leiomyosarcoma (LEIS).

**Figure 5:**
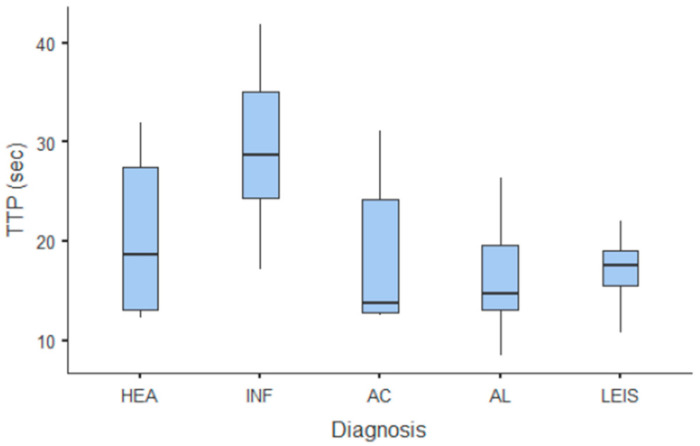
Box and whisker plot of the time to peak (TTP) for healthy (HEA), inflammatory (INF), adenocarcinoma (AC), lymphoma (AL), and leiomyosarcoma (LEIS).

**Figure 6:**
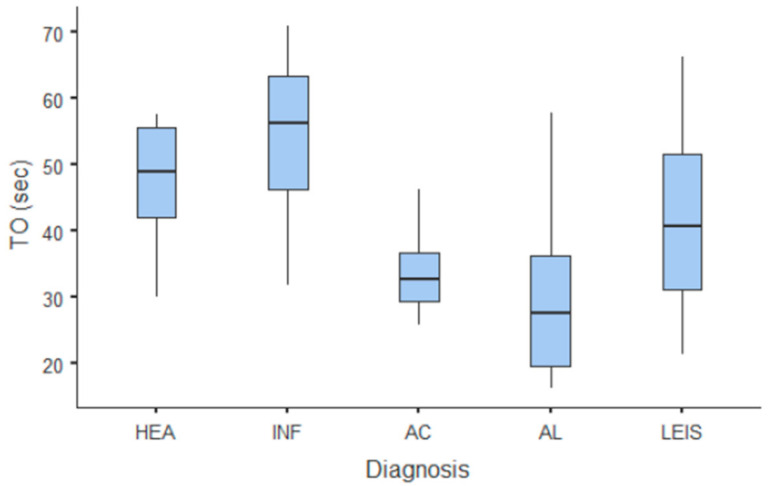
Box and whisker plot of the time to fall (TO) for healthy (HEA), inflammatory (INF), adenocarcinoma (AC), lymphoma (AL), and leiomyosarcoma (LEIS).

**Figure 7:**
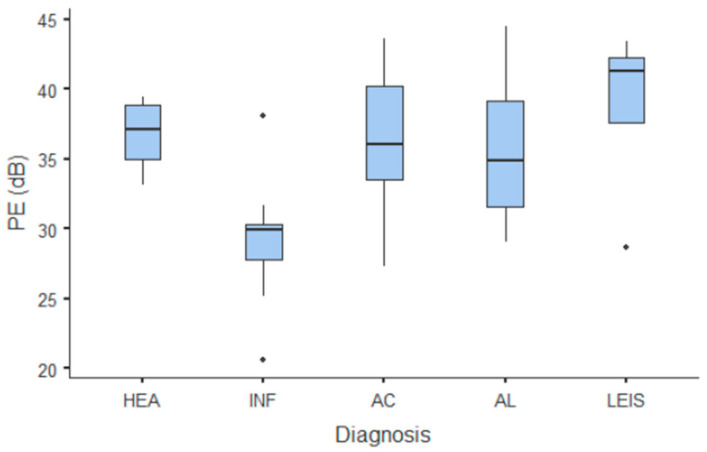
Box and whisker plot of the peak enhancement (PE) for healthy (HEA), inflammatory (INF), adenocarcinoma (AC), lymphoma (AL), and leiomyosarcoma (LEIS).

**Figure 8:**
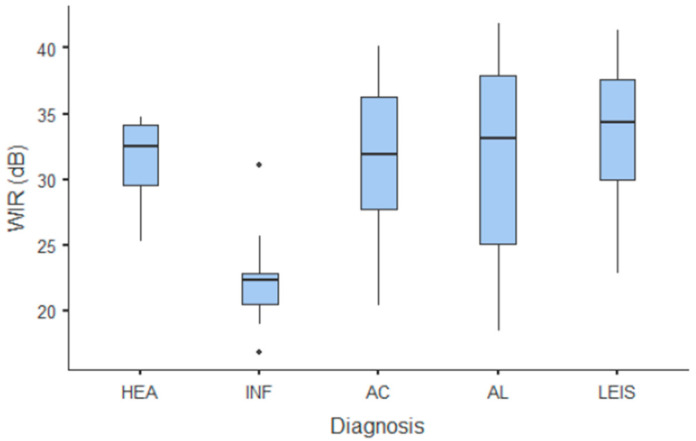
Box and whisker plot of the wash-in rate (WiR) for healthy (HEA), inflammatory (INF), adenocarcinoma (AC), lymphoma (AL), and leiomyosarcoma (LEIS).

**Figure 9:**
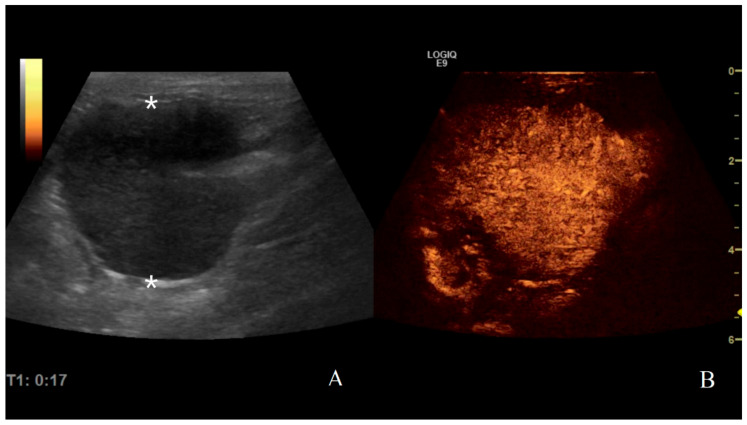
Gastric lymphoma in a dog: (**A**) B-mode image of a severe focal transmural hypoechoic mass of 24 mm (between asterisks) with absent layers definition; (**B**) contrast enhanced ultrasound (CEUS)examination of the same gastric mass during the peak enhancement (17 s) of the time-intensity curve.

**Figure 10:**
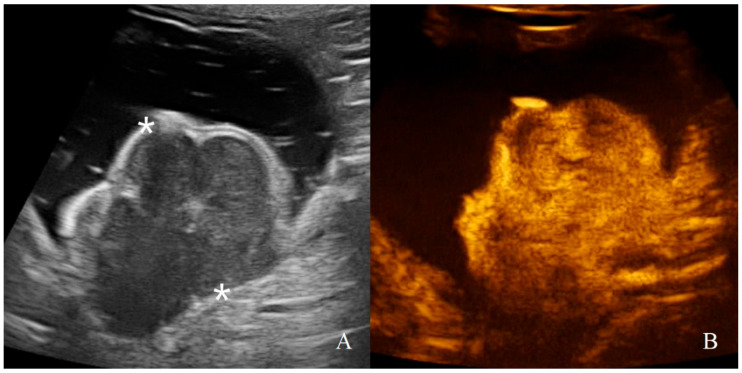
Gastric leiomyosarcoma in a dog: (**A**) B-mode image of a focal hyperechoic wall mass protruding into the gastric lumen (19.3 mm between asterisks) with no layers definition; (**B**) CEUS examination of the same dog during the peak enhancement of the time-intensity curve.

Summary statistics of the CEUS quantitative parameters for the different groups are reported in Table 3. Multiple comparison graphs of AT, TTP, TO, PE, and WiR, in which statistically significant differences were found between groups, are reported in Figure 4, Figure 5, Figure 6, Figure 7 and Figure 8, respectively. All the dogs included receive three consecutive contrast injections. In most cases (36/41 dogs), the second injection was used to perform CEUS quantitative analysis. The third injection was used only in cases where the quality of the second scan was considered insufficient due to excessive gas and/or motion artifacts (5/41 dogs: 1 HEA; 2 INF; 1 AC; 1 AL). However, the authors noted no significant differences in parameters variability between measurements taken during the second and third injection within the same groups. Since data collected for time and intensity values were normally distributed, the differences between groups were calculated by means with ANOVA. The INF is the only group that showed significant differences: dogs affected by gastritis have, in general, a lower (*p* < 0.05 for PE and WiR) and delayed (*p* < 0.05 for AT, TTP, and TO) contrast uptake compared to that of AC, AL (Figure 9), and LEIS (Figure 10). No differences were evident between any other group for all the other intensity and time parameters.

**Figure 11:**
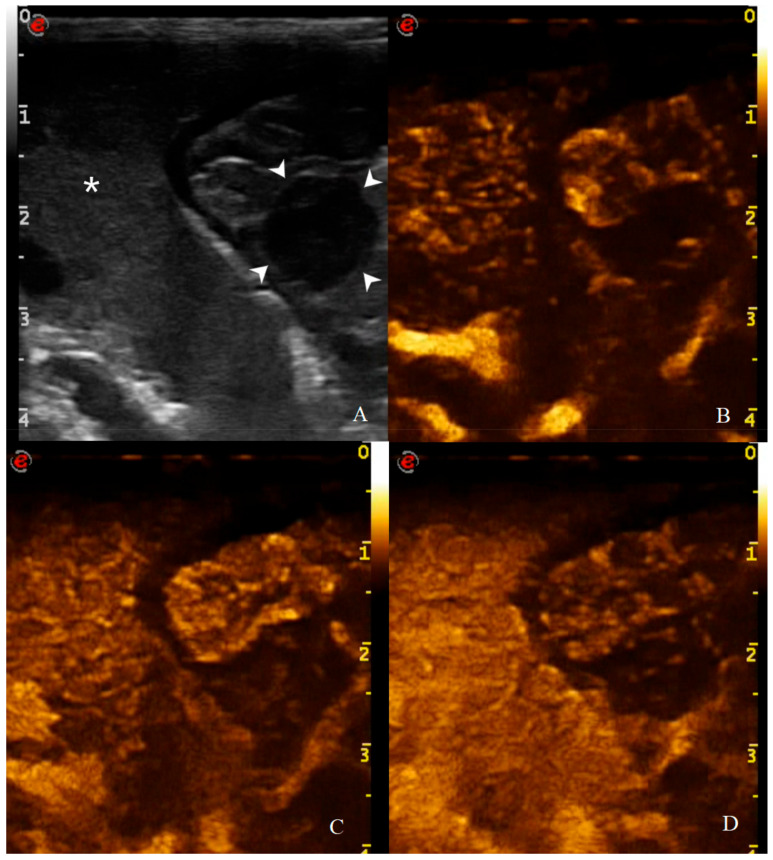
CEUS examination of the same dog during the peak enhancement of the time-intensity curve. CEUS enhancement pattern in a canine adenocarcinomas: (**A**) before contrast medium (CM) injection (0 s), the gastric mass (14 mm—arrowheads) and the adjacent liver parenchyma (asterisk) are visualized; (**B**) after CM injection (13 s), uptake occurs at the same moment at gastric wall and liver and the mass is classified as isoenhancing; (**C**) at peak enhancement time, contrast uptake is higher at the lesion margins and poor at its center and enhancement homogeneity is classified as heterogeneous; (**D**) in wash out phase (23 s), the enhancement decreased at gastric wall but persist in the adjacent liver.

Qualitative assessment and enhancement pattern (Figure 11) of CEUS were performed and reported in Table 4. Significant differences were found in enhancement homogeneity between HEA vs. AC (*p* = 0.015), INF vs. AC (*p* = 0.004), and INF vs. LEIS (*p* = 0.038). No other qualitative parameter showed statistical significance. The only groups that showed “comb teeth-like vessels” pattern were AC (2/8) and the metastatic hemangiosarcoma (1/1).

## 4. Discussion

Canine gastric disorders have shown to have different origin and highly variable US characteristics. Among the tumors, AC and AL were found to be the most frequent in dogs in our series, with an identical prevalence (30.7% each), differing from what is reported in the literature, in which a much higher prevalence is described for AC than the other canine gastric neoplasia [1,7,8,9,10,11]. Other malignancies less represented in our series include LEIS (15.3%), GIST (7.7%), sarcoma, and hemangiosarcoma (3.8% each). The only benign tumor found in this study was LEIM (7.7%).

The standard US features of canine healthy and pathological gastric wall are reported in the veterinary literature [3,22,45,46,47]. Wall thickness is the main quantitative parameter considered in the US evaluation of the gastric wall on B-mode. In our group of HEA dogs, the average wall thickness (mean 4 mm) combined with the absence of layers alteration, appears to be in the range reported in the literature (3–5 mm) [3,16,22,47]. Focal or diffuse severe wall thickening, associated with loss of layering, is highly suggestive of gastric neoplasia [22,46,48]; although, also inflammatory disorders, like gastritis or enteritis, may cause segmental or diffuse wall thickening with reduced or loss of layering definition [46,48]. In our population of dogs suffering from gastric disorders, the INFs have been shown to have an average slightly thickened gastric wall (mean 6.2 mm) associated with normal (5/9) or reduced (4/9) layers definition. Otherwise, all the neoplastic cases showed a marked wall thickening, exceeding 12 mm in the 92.3% of the dogs. Furthermore, in our series, the absence of normal layers definition was the other main findings in case of gastric neoplasia since 23/26 dogs with a wall mass presented this feature. Only 2/8 gastric AC showed a pseudo-layering, despite this aspect being reported as typical of this histotype: 16/17 dogs affected by gastric AC shown a pseudolayering aspect in another series described by Penninck et al. (1998) [22]. As for lesion echogenicity, no specific aspect was found for any of the tumors histotypes since neoplastic masses may have a highly variable appearance even within the same groups. However, based on those two criteria (wall thickness and layers definition), no statistically significant differences were found between the different groups of tumors. Gastritis, on the other hand, showed significant differences based on thickness and layers definition when compared with AC, AL, and LEIS. Furthermore, also the other B-mode parameters considered did not show specific differences between AC, AL, and LEIS but only between these and the HEA and INF dogs (Table 2).

These findings confirm what has been reported in veterinary literature for canine gastrointestinal diseases through the years, with gastritis usually appearing on B-mode US as a normal/mild thickening with preserved/reduced layers definition, while gastric tumors showing severe thickening with loss of wall layering and altered echogenicity and echotexture [11,45,46,48,49]. Thickening localization, lesion echogenicity, regional lymph nodes appearance, presence of local steatitis and/or effusion have underlined and reconfirmed how canine gastric neoplasms may have variable features on B-mode US. These results confirm, once again, the need to always perform cytological and/or histological examinations to characterize the neoplastic gastric lesions as reported in veterinary literature [1,11,16,50].

All 43 dogs that underwent CEUS examination showed no adverse reactions to the CM with stable respiratory and cardiovascular parameters during the examination time, confirming this technique as safe and well tolerated [23].

The CEUS quantitative time and intensity parameters have highlighted specific characteristics only for INF when compared to neoplasms groups: gastritis generally seems to have longer wash-in and wash-out times if compared to AC, AL, and LEIS; except for RT for which no difference was found between any of the considered groups. Likewise, maybe as a direct consequence, the intensity values (PE for AC and LEIS; WiR for all the neoplasm) of contrast uptakes are substantially lower and delayed than in tumors groups, but more persistent over time. No specific and significant characteristics were highlighted for AC, AL, and LEIS between each other: CEUS parameters overlapped both in terms of time uptake and signal intensity. Furthermore, also the HEA control group seems to have overlapping values with INF; AC, AL, and LEIS, which therefore does not allow to highlight significant differences useful to discriminate it from the pathological groups. These results are partially similar to what has been reported and published for CEUS parameters in feline gastric diseases [41]. In fact, high-grade feline gastric lymphomas showed higher intensity values (mean 38.99 dB for PE and 34.91 for WiR) if compared to gastritis and low-grade lymphoma (means around 30 dB for PE and around 25 for WiR), but similar to healthy gastric wall; for time values, high-grade lymphomas show lower time values only in washout phase when compared to healthy, inflammatory, or low-grade lymphoma [41]. Furthermore, as already described in [41], comparing our results with that reported in human medicine by Xue et al. (2017), the CEUS quantitative findings seem to indicate a different vascular kinetics also for canine gastric disorders from those of human patients: malignant lesions are reportedly characterized by lower values of contrast uptake (PE) and a delayed washout if compared to those of gastritis [43].

To further assess the vascular component on CEUS examination, qualitative analysis of MDC uptake at gastric wall was also performed (Table 4). Gastric wall neoplasms showed a certain variability in terms of enhancement degree and homogeneity: ACs show to be predominantly hypo/isoenhancing compared to the adjacent hepatic parenchyma (8/8) with heterogeneous uptake of MDC (7/8); AL and LEIS instead show to have a more variable vascular behavior on CEUS examination, without a significant prevalence in terms of enhancement degree or homogeneity. Furthermore, the comb teeth like vessels pattern were observed only in one case of AC (1/8) and in the dog with gastric hemangiosarcoma. These data partially differ from what has been reported in cats, in which high grade lymphomas showed variable enhancement degree (5/10 hyper and 5/10 hypo/iso enhancement) and homogeneous enhancement (7/10) with the presence of comb teeth-like vessels (7/10) [41].

The potential limitations of this study include the difficulty in gastric wall measurement for the CEUS parameter evaluation program, especially if no significant thickenings or masses are present, due to the limited space in which the ROI can be drawn within the gastric wall; furthermore, gas reverberation artifacts and breathing movement may alter the measurements at the ROI site. For this reason, the CEUS examinations were performed in anesthetized patients to minimize these artifacts. This choice may represent a further potential limitation since anesthetic drugs may cause alteration on gastric wall perfusion. However, since all patients underwent the same protocol, this variable should not be significant and should not compromise interpretation of collected data. Other study limitations relate to the animals included: the lack of cyto- or histopathological examination of the gastric wall for the HEA control group, in which, however, the absence of clinical, laboratory, and ultrasonographic signs compatible with gastric disease was confirmed both at the first clinical evaluation, and at the follow-up after 12 months.

## 5. Conclusions

The results of this study demonstrate how gastric inflammation and neoplasms can show different B-mode and CEUS features on dogs. On B-mode dogs affected by gastritis usually showed a normal to mild increase wall thickness, with normal to reduce layers definition, with no alteration of adjacent structures. Gastric neoplasia, instead, usually presents a gastric focal mass with variable echogenicity, possible involvement of regional lymph nodes (AC and AL), and presence of steatitis (AC). On CEUS examination, neoplasm show a higher and faster wash in if compared to that of gastritis. B-mode and CEUS assessment may be useful in the evaluation of canine gastric disorders in distinction between gastritis and gastric neoplasms, even if there are no specific features able to discriminate between the different tumors histotypes. Future studies could focus on further evaluations of the different canine gastrointestinal tumors histotypes using other US techniques such as elastosonography.

## Figures and Tables

**Table 1:** Wall thickness B-mode evaluation for healthy (HEA), inflammatory (INF), adenocarcinoma (AC), lymphoma (AL), and leiomyosarcoma (LEIS) reported as means with standard deviations (±SD) and ranges.

Wall Thickness (mm)	HEA	INF	AC	AL	LEIS	*p*-Value
Mean	4.06 ^a^	6.27 ^a^	20.1 ^b^	22.1 ^b^	27.06 ^b^	<0.001
±SD	1.08	0.94	4.74	12.5	7.53	
Range	3.17–6	4.8–7.6	14–28	7.3–39.6	19.3–37.3	

^a,b^ Values with different superscript letters differ significantly (*p* < 0.05).

**Table 2:** Number of cases (*n*) and percentage (%) showing qualitative features at B-mode ultrasound for healthy (HEA), inflammatory (INF), adenocarcinoma (AC), lymphoma (AL), and leiomyosarcoma (LEIS).

B-Mode Qualitative Variables	HEA (*n* = 6) %	INF (*n* = 9) %	AC (*n* = 8) %	AL (*n* = 8) %	LEIS (*n* = 4) %	*p*-Value
**Layer definition**						<0.001
Normal	100	55.6	0	0	0	
Reduced	0	44.4	0	0	0	
Absent	0	0	75	100	100	
Pseudolayering	0	0	25	0	0	
**Thickening**						0.010
Absent	100	55.6	0	0	0	
Focal	0	0	87.5	75	100	
Diffuse	0	44.4	12.5	25	0	
**Lesion echogenicity**						<0.001
Iso/Hypoechoic	NA	NA	25	12.5	0	
Hyperechoic	NA	NA	50	25	50	
Mixed	NA	NA	25	62.5	50	
**Regional lymph nodes**						0.006
Normal	100	100	50	37.5	100	
Enlarged	0	0	50	62.5	0	
Steatitis						0.006
Present	0	0	75	37.5	25	
Absent	100	100	25	62.5	75	
Effusion						0.650
Present	0	0	12.5	12.5	0	
Absent	100	100	87.5	87.5	100	

**Table 3:** Quantitative parameters reported as mean values of contrast enhanced ultrasound (CEUS) examination for healthy (HEA), inflammatory (INF), adenocarcinoma (AC), lymphoma (AL), and leiomyosarcoma (LEIS). Parameters considered include arrival time (AT), rising time (RT), time to peak (TTP), time to fall (TO), peak enhancement (PE) and wash-in rate (WiR).

CEUS Quantitative Features.	HEA (*n* = 6)	INF (*n* = 9)	AC (*n* = 8)	AL (*n* = 8)	LEIS (*n* = 4)	*p*-Value
AT (s)	14.1	21.6	13.5	10.8	9.93	0.007
RT (s)	6.43	7.24	4.84	5.71	7.02	0.410
TTP (s)	20.05	28.8	18.3	16.5	30.7	0.040
TO (s)	47.2	54.6	33.5	30.07	42.2	0.010
PE (dB)	36.8	29.3	36.4	35.8	38.6	0.030
WiR (dB)	31.4	22.4	31.6	31.4	33.2	0.008

**Table 4:** Qualitative parameters reported as mean values of CEUS examination for healthy (HEA), inflammatory (INF), adenocarcinoma (AC), lymphoma (AL), and leiomyosarcoma (LEIS).

CEUS Qualitative Variables	HEA (*n* = 6) %	INF (*n* = 9) %	AC (*n* = 8) %	AL (*n* = 8) %	LEIS (*n* = 4) %	*p*-Value
**Enhancement degree**						0.93
Hypo/Isoenhancing	66.7	88.9	100	75	50	
Hyperenhancing	33.3	11.1	0	25	25	
Non-enhancing	0	0	0	0	25	
**Enhancement homogeneity**						<0.001
Homogeneous	100	100	12.5	50	25	
Heterogeneous	0	0	87.5	50	50	
No uptake	0	0	0	0	25	
**Comb teeth-like vessels**						0.73
Present	0	0	12.5	0	0	
Absent	100	100	87.5	100	100	

## Data Availability

The data presented in this study are available on request from the corresponding author. The data are not publicly available due to privacy.
